# Second generation sequencing and morphological faecal analysis reveal unexpected foraging behaviour by *Myotis nattereri* (Chiroptera, Vespertilionidae) in winter

**DOI:** 10.1186/1742-9994-11-39

**Published:** 2014-05-09

**Authors:** Paul R Hope, Kristine Bohmann, M Thomas P Gilbert, Marie Lisandra Zepeda-Mendoza, Orly Razgour, Gareth Jones

**Affiliations:** 1 School of Biological Sciences, University of Bristol, Woodland Road, Bristol BS8 1UG, UK; 2 Ecological Consultancy Services Ltd, Longdown, Salisbury Road, Shootash SO51 6GA, UK; 3 Section for Evolutionary Genomics, Centre for GeoGenetics, Natural History Museum of Denmark, Copenhagen, Denmark

**Keywords:** Echolocation, Ecosystem services, Hibernation, Illumina MiSeq, Metabarcoding, Molecular diet analyses, Natterer’s bat, Sensory ecology, Winter diet

## Abstract

**Background:**

Temperate winters produce extreme energetic challenges for small insectivorous mammals. Some bat species inhabiting locations with mild temperate winters forage during brief inter-torpor normothermic periods of activity. However, the winter diet of bats in mild temperate locations is studied infrequently. Although microscopic analyses of faeces have traditionally been used to characterise bat diet, recently the coupling of PCR with second generation sequencing has offered the potential to further advance our understanding of animal dietary composition and foraging behaviour by allowing identification of a much greater proportion of prey items often with increased taxonomic resolution. We used morphological analysis and Illumina-based second generation sequencing to study the winter diet of Natterer’s bat (*Myotis nattereri)* and compared the results obtained from these two approaches. For the first time, we demonstrate the applicability of the Illumina MiSeq platform as a data generation source for bat dietary analyses.

**Results:**

Faecal pellets collected from a hibernation site in southern England during two winters (December-March 2009–10 and 2010–11), indicated that *M. nattereri* forages throughout winter at least in a location with a mild winter climate. Through morphological analysis, arthropod fragments from seven taxonomic orders were identified. A high proportion of these was non-volant (67.9% of faecal pellets) and unexpectedly included many lepidopteran larvae. Molecular analysis identified 43 prey species from six taxonomic orders and confirmed the frequent presence of lepidopteran species that overwinter as larvae.

**Conclusions:**

The winter diet of *M. nattereri* is substantially different from other times of the year confirming that this species has a wide and adaptable dietary niche. Comparison of DNA derived from the prey to an extensive reference dataset of potential prey barcode sequences permitted fine scale taxonomic resolution of prey species. The high occurrence of non-volant prey suggests that gleaning allows prey capture at low ambient temperatures when the abundance of flying insects may be substantially reduced. Interesting questions arise as to how *M. nattereri* might successfully locate and capture some of the non-volant prey species encountered in its faeces. The consumption of lepidopteran larvae such as cutworms suggests that *M. nattereri* eats agricultural pest species.

## Background

The onset of winter with associated decreases in ambient temperature leads to increased energetic challenges for mammals in many temperate regions. Small insectivorous mammals face two key challenges. First, their high surface area-to-volume ratio renders them vulnerable to high levels of heat loss and energy expenditure at low temperatures [1,2]. Second, they face a reduction in available prey because reduced temperatures reduce arthropod abundance and activity [3,4]. Heterothermic mammals such as bats, cope with these challenges using hibernation, that is, by lowering their body temperature, metabolic rate and therefore energetic requirements over extended periods of time [2].

All hibernators arouse periodically, probably to meet behavioural or physiological requirements that are brought about by their reduced metabolism [5]. Studies on hibernating bats in cold continental locations (where winter temperatures rarely rise above freezing) show that although arousals do occur, they are relatively infrequent [6-8]. In contrast, bats arouse from hibernation more frequently in locations where winters are mild [9-13]. The relatively high energetic costs of winter arousal [2,14] will dictate that periods of euthermia are kept to the minimum necessary.

To obtain a net energetic gain from foraging, insect prey need to be available in sufficient quantities to offset the costs of activity. Insect flight activity declines dramatically as ambient temperature drops below 6-10°C [3,15], but some arthropods remain active on the ground or amongst foliage at temperatures below those suitable for flight [3,16,17]. The sensory ecology of a predator, and therefore the range of foraging techniques that it may utilise, will play an important role in its ability to exploit potential opportunities for feeding in winter [17,18]. For example, foraging may remain profitable for bats that glean prey from vegetation (gleaning bats) even during nights when ambient temperatures drop below the threshold for insect flight [13].

### Evidence for winter foraging

In temperate locations where winter temperatures are relatively mild, bat activity outside of the hibernacula occurs frequently during winter. For instance, in New South Wales, Australia, tree-roosting bats, *Nyctophilus geoffroyi* and *N. gouldi,* were active for longer on warmer winter nights suggesting occasional winter foraging [11]. Similarly, in the south of England, feeding buzzes (increase in echolocation pulse rate as a bat attempts to catch prey) were recorded during winter from foraging pipistrelles (*Pipistrellus* spp*.*) [19] and noctules (*Nyctalus noctula*) [20], and a single Natterer’s bat (*Myotis nattereri*) was radio tracked foraging outside of the hibernaculum over ten winter nights in the south of England [13]. Furthermore, studies on the winter diet of rhinolophid bats confirmed that these species feed throughout the winter in the United Kingdom [21,22] and continental Europe [23]. The majority of arousals [10,13] and peaks in subsequent activity [24,25] are synchronised around sunset, when insects will be most abundant [11,26]. Activity is also more likely on milder nights when insect abundance and activity is greater [27]. In contrast, arousals [8] and subsequent activity [28] in cold continental climates do not appear to be synchronised with zeitgebers such as photoperiod or temperature.

Until now, the winter diet of vespertilionid bats in locations with mild temperate climate has been largely overlooked, but studies of the activity and diet of winter flying northern bats (*Myotis septentrionalis*) and little brown bats (*Myotis lucifugus*) in Indiana, USA, demonstrated that these bats did not feed during the winter [29,30]. Dehydration or the selection of more suitable roosting location may be a driving force behind the winter flights of bats in such cold continental regions [31].

### Studies of trophic ecology

Traditionally, microscopic analysis has been used to identify prey insects remains in faecal pellets produced by insectivorous bats [32]. This technique is complicated by the bats’ tendency to rapidly and thoroughly chew their prey and spit out hard parts, resulting in limited taxonomic resolution of the diet [33].Consequently, during the last five years, molecular diet analyses, which can yield great taxonomic resolution of prey if the reference database is comprehensive, have increased in popularity. Initially, such molecular studies of bat diet were typically conducted by PCR amplification of prey DNA followed by molecular cloning so as to enable subsequent Sanger sequencing of the composite prey sequences (e.g. [34,35]). Subsequently, however, the introduction of second generation sequencing approaches such as the GS FLX [36,37] and Ion-torrent PGM [38-40] increased sequencing power while decreasing the cost of metabarcoding, and were therefore rapidly adopted for bat diet analyses. To our knowledge, at the time of writing, the current study is the first time the Illumina MiSeq platform is used as a data generation source for bat dietary analyses.

In this study, we used both Illumina MiSeq-based second generation sequencing and microscope-based morphological analyses to identify the constituent prey in faecal pellets from *Myotis nattereri* sensu stricto [41] collected during two winters. The overall aim was to investigate the winter diet of *M. nattereri* at this hibernation site, while comparing the scope of the two analytical techniques. We find that quantitative representation of diet scored by morphological and molecular analyses are significantly positively correlated at the order level, and that *M. nattereri* feed on a high proportion of non-flying invertebrates in winter, indicating a gleaning foraging strategy. We discuss the potential challenges that *M. nattereri* may face in detecting non-flying invertebrate prey items.

## Results

Amplified Cyt b sequences were successfully obtained from 73 (81%) of the 90 extracted faecal samples. A match to bat species (using the criteria set out in Methods) was obtained for 71 (79%) of these, with 62 identified as *Myotis nattereri* sensu stricto and the remainder as *Myotis daubentonii* (GenBank accession numbers: KJ719267- KJ719283)*.* Samples that yielded no positive match to a single bat species were discarded. All subsequent DNA and morphological analyses were conducted using only the *M. nattereri* samples. Of these 62 analysed droppings, 25 (40%) were collected in December, 21 (34%) in January and February and 16 (26%) during March. Each period included samples collected during both winters (2009–10 and 2010–11) of the study.

### Morphological analysis

Microscopic analysis revealed arthropod fragments from seven taxonomic orders (Table 1). When all samples were pooled (*n* = 62) representing both winters, Lepidoptera was the most frequently occurring order measured by% occurrence (%O) (Table 1), though a high proportion of these remains were attributed to lepidopteran larvae. Araneae was the next most frequently encountered order in pellets, followed by Isopoda and Diptera. Coleoptera, Hymenoptera and Hemiptera were less frequently encountered over the entire winter period. By proportion of pooled data (%F), 67.9% of taxa recorded were either non-volant invertebrates or identified as being in a non-volant stage of their life cycle (i.e. lepidopteran larvae).

**Table 1:** **Percentage of prey orders encountered within *M.nattereri *** **faecal pellets**

**Order**	**Morphological**		**Molecular**	
	**% O**	**% F**	**% O†**	**% F†**
Araneae	59.7	28.2	56.5	17.9
Isopoda	32.3	15.3	90.3	28.7
Lepidoptera	88.7	42.0	98.5	31.3
Hymenoptera	1.6	0.7	3.2	1.0
Hemiptera	1.6	0.8	-	-
Coleoptera	3.2	1.5	14.5	4.6
Diptera	24.2	11.5	51.6	16.4

Percentage occurrence (%O) of prey orders in *M. nattereri* faecal pellets gathered in Greywell tunnel during winters (December-March) 2009–10 and 2010–11. Also shown are the proportions (%F) of prey orders in the diet. † OTUs identified to species using BOLD then assigned to individual pellets.

The percentage frequency (%F) of adult Lepidoptera was highest during January and February (26%), while the proportion of lepidopteran larvae increased as winter progressed from 18% in December, to 26% in January/February up to 34% in March. The percentage frequency (%F) of Diptera was highest during December (20%), and then dropped to its lowest value during January and February (2%). The proportion (%F) of non-volant invertebrates was at its highest during January/February (70%).

### Molecular analysis

A total of 99 Operational Taxonomical Units (OTUs) were identified in the 62 *Myotis nattereri* faecal droppings sequenced on the Illumina MiSeq platform. The assigned OTUs were divided into three main groups: Arachnida, Insecta, and Malacostraca; which contained 297, 968, and 897 collapsed haplotypes respectively. Furthermore, 1,834 collapsed haplotypes could be assigned to species level. The number of prey taxa (OTUs) per faecal sample ranged between one and 21 with a mean of 8.1 (SD ±  3.9). We successfully identified arthropod DNA from six taxonomic orders (Table 1), and were able to assign OTUs to 43 arthropod species (and an additional six OTUs to one of two species) from 24 families (Table 2). DNA analysis failed to identify only one order (Hemiptera) that had been identified through morphological analysis.

**Table 2:** **List of prey identified within *M.natterei *** **faecal pellets through high-throughput sequencing**

**Order**	**Family**	**Species**	**Confidence**	**Sequence**	**Number of faecal pellets in which found**
			**level**	**Similarity %**	
Lepidoptera	Geometridae^1^	*Agriopis marginaria*	2	100	4
		*Alcis resplandata*	2	100	2
		*Operophtera brumata*	1	100	19
	Noctuidae^1^	Unknown	4	100	7
		*Anaplectoides prasina*	2	100	1
		*Apamea epomidion*	1	100	1
		*Apamea crenata/epomidion*	3	100	2
		*Conistra vaccinii/ligula*	3	98.72	2
		*Diarsia rubi*	2	100	2
		*Noctua sp.*	1	100	3
		*Noctua janthe*	3	100	1
		*Noctua pronuba*	3	100	39
		*Omphaloscelis lunosa*	1	100	2
		*Orthosia incerta*	2	100	6
		*Phlogophora meticulosa*	2	100	1
		*Xestia c-nigrum*	2	100	12
		*Xestia sextrigata*	2	100	8
		*Xestia triangulum*	2	100	13
		*Xestia xanthographa*	2	100	50
	Torticidae	*Tortricodes alternella*	1	100	9
	Ypsolophidae	*Ypsolopha ustella*	2	100	1
Diptera	Calliphoridae	*Pollenia rudis*	1	100	4
		*Pollenia pediculate*	1	99.36	1
		*Pollenia sp.*	1	100	14
	Chironomidae	*Prodiamesa olivacea*	1	98.72	1
	Culicidae	*Culex torrentium/pipiens*	3	100	1
	Muscidae	*Eudasyphora cyanicolor*	1	99.36	1
		*Musca autumnalis*	1	100	2
		*Phaonia tuguriorum*	1	100	1
	Scathophagidae	*Scathophaga stercoraria*	1	99.28	6
	Tachinidae^2^	*Ramonda spathulata*	1	100	7
Coleoptera	Cantharidae	*Cantharis cryptica*	1	100	1
		*Cantharis decipiens*	1	100	1
		*Cantharis livida/pellucida*	3	100	1
	Carabidae	*Notiophilus biguttatus*	1	99.36	1
	Curculionidae	*Sitona lineatus/suturalis*	3	100	5
Hymenoptera	Tenthredinidae	*Tenthredopsis sp.*	4	99.36	1
Isopoda	Philosciidae	*Philoscia muscorum*	1	100	59
Araneae	Anyphaenidae	*Anyphaena accentuate*	1	100	3
	Araneidae	*Cyclosa conica*	1	99.36	1
		*Zygiella x-nonata*	1	100	2
	Linphilidae	*Linyphia hortensis*	1	100	3
		*Neriene peltata*	1	100	2
		*Neriene montana*	1	100	2
	Lycosidae	*Pardosa sp.*	1	100	1
	Philodromidae	*Philodromus aureoles*	1	100	1
		*Philodromus cespitum*	1	100	1
		*Philodromus dispar*	2	99.35	1
	Pisauridae	*Pisaura mirabilis*	1	100	2
	Tetragnathidae	*Metellina segmentata*	1	100	10
	Theridiidae	Unknown	1	100	1
		*Phylloneta impressa*	1	100	2
		*Paidiscura pallens*	1	100	2
	Thomisidae	*Diaea dorsata*	1	100	3
		*Xysticus sp.*	1	100	2

List of prey identified in 62 *M. nattereri* faecal pellets collected within Greywell Tunnel between December–March 2009–10 and 2010–11. Confidence levels follow Razgour et al. [37] and are based on the BOLD identification system, whereby confidence level 1 = solid match to one species or genus (>98.5%); level = 2 match to more than one species (98.5%), only one of which was a UK species; level 3 = matched to two UK species of the same family (>98.5%) and level 4 = match to several species of different genera, or to reference sequences only identified to family (>98%). Superscript indicates families where species are known to possess tympanate hearing organs ^1^[42], ^2^[43].

The percentage frequency of arthropod orders shows a similar seasonal trend to the morphological results. The proportion (%F) of Lepidoptera remained relatively stable over the winter (December: 30%; January/February: 32%; March: 33%), while the proportion (%F) of arachnids decreased in early spring (December: 23%; January/February: 20%; March: 6%). The proportion (%F) of Diptera decreased from December (18%) to 13% in January/February then increased again during March (18%).

Isopods comprised a substantial part of the diet throughout the sampling period and were recorded in all but three (*n* = 59) of the faecal samples. The large numbers of isopods found conflicts with the results from the morphological analysis where this order was recorded in less than one-third (*n* = 20) of all faecal samples. Coleoptera, Hymenoptera and Hemiptera formed a very small component part of the winter diet of *M. nattereri* making up <4% of morphological and <6% of molecular dietary composition.

**Figure 1:**
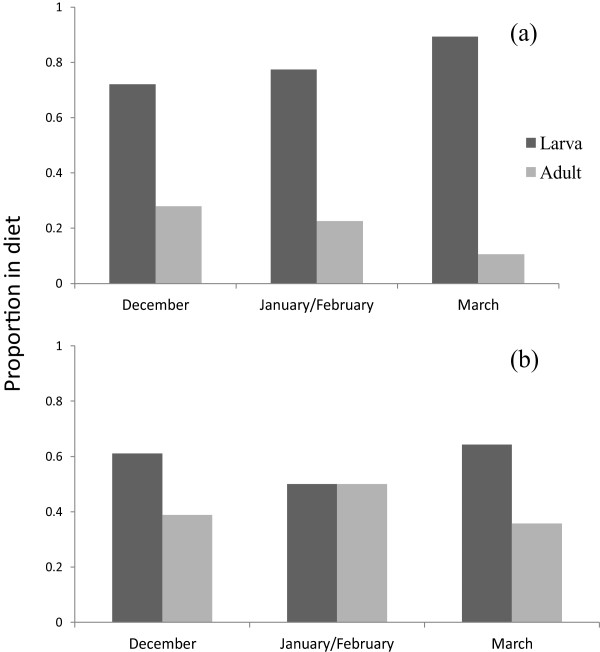
**Percent frequency of lepidopteran adults and larvae in the diet of *M. nattereri *during three winter periods using data from 2009–10 and 2010–11 combined. a)** Results from molecular analysis, whereby adults or larvae are determined by the proportion of OTUs allotted to either adult or larval stages according to the known phenology of species encountered. **b)** Results from morphological analysis where adult or larvae are determined from lepidopteran body fragments recovered from faecal pellets.

The molecular results show that the percent frequency (%F) of lepidopteran larvae identified was greater than that of adults in all winter months (Figure 1a). Morphological findings (Figure 1b) suggest a higher proportion of larvae in December and March compared to adults.

**Figure 2:**
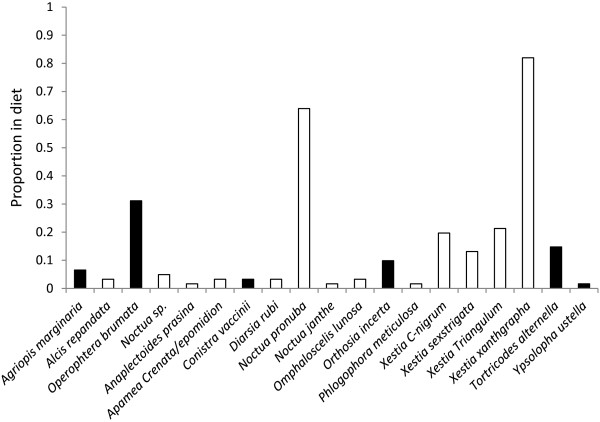
**The proportion of Lepidoptera (%F) by species in the winter diet of *M. nattereri *from DNA analysis of faecal pellets collected from within Greywell Tunnel in December-March 2009–10 and 2010–11 combined.** Open bars indicate species predicted to overwinter as larvae and black bars indicate those species that overwinter as adults [44,45].

The proportion of Lepidoptera (%F) by species (Figure 2) shows that the putative larval sequences identified were dominated by the square spot rustic, *Xanthia xanthographa,* and the large yellow underwing, *Noctua pronuba*.

**Figure 3:**
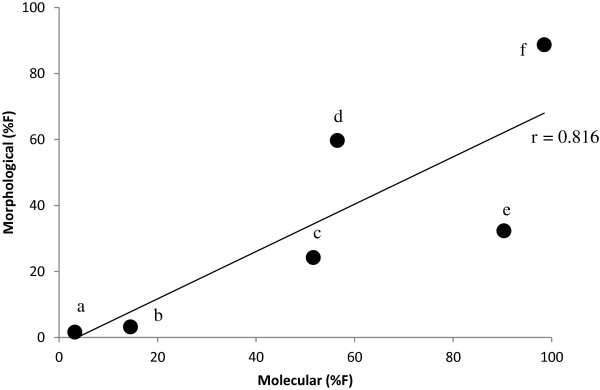
**Comparison between percentage frequency (%F) of prey orders in faecal pellets found by using morphological and molecular analyses (OTUs identified to species using BOLD then assigned to individual pellets).** Labels identify the taxonomic orders as follows, **a)** Hymenoptera, **b)** Coleoptera, **c)** Diptera, **d)** Araneae, **e)** Isopoda and **f)** Lepidoptera.

We compared the percentage frequency (%F) of prey orders in faecal pellets between the morphological and molecular data (Figure 3). Although Isopoda were proportionately more frequent in the molecular analysis, there was nevertheless a significant positive correlation in the occurrence of prey orders described by the two methods (*r* = 0.816, *n* = 6, *P* <0.05).

## Discussion

Using second generation sequencing approaches to assess diet can be a very powerful tool [36,46-49], also see review by Pompanon et al. [50]. These approaches can detect prey items that are not visible under a microscope, and if the reference database is comprehensive, they can be used to identify prey species at a much finer taxonomic level than morphological analysis [34]. If the reference database is not comprehensive, however, the difference between morphological and molecular analyses in the taxonomic level of prey identification becomes less pronounced and as with morphological analysis, the results from the molecular analysis will have to be presented at order or family level while species numbers will have to be inferred by OTUs [36]. Despite the data generation power of second generation sequencing and the ability to present results at a much higher taxonomic resolution, it does have its shortcomings compared to microscopic analysis. For example, molecular approaches are unable to identify different life stages of prey, as the DNA of e.g. a moth will be the same no matter if the moth is a larva or an adult. Also, identification of the noctuid parasite, the tachinid fly (*Ramonda spathula*) (Table 3) in 11.3% of analysed pellets shows that second generation sequencing can even detect internal prey parasites. For both parasites and secondary predation, molecular (and to a lesser extent morphological) analyses of diet can make it difficult to discern between prey, which were eaten directly and prey that were consumed because they were in turn eaten by prey [51] or because they parasitised the prey.

**Table 3:** **Phenology of lepidopteran prey species found in the winter diet of *M. nattereri***

**Family**	**Species**	**Flight season**	**Life cycle**
Geometridae	*Agriopis marginaria*	February- April	Larva April- June
	*Alcis resplandata*	June- August	Overwinters as larva
	*Operophtera brumata*	October- January	Larva April- June
Noctuidae	*Anaplectoides prasina*	June- July	Overwinters as larva
	*Apamea epomidion*	June- July	Overwinters as larva
	*Apamea crenata/epomidion*	June- July	Overwinters as larva
	*Conistra vaccinii/ligula*	September- May	Larva April- June
	*Diarsia rubi*	May- June, August- September	Overwinters as larva
	*Noctua pronuba/janthe*	June- November	Overwinters as larva
	*Omphaloscelis lunosa*	August- October	Overwinters as larva
	*Orthosia incerta*	March- May	Larva April- June
	*Phlogophora meticulosa*	May- October	Overwinters as larva,
	*Xestia c-nigrum*	May- June, August- October	Overwinters as larva
	*Xestia sextrigata*	July- August	Overwinters as larva
	*Xestia triangulum*	June- August	Overwinters as larva
	*Xestia xanthographa*	June- October	Overwinters as larva
Torticidae	*Tortricodes alternella*	January- April	Larva, May
Ypsolophidae	*Ypsolopha ustella*	Throughout year	Larva April- June

Phenology of lepidopteran prey species found in the winter diet of *M. nattereri*, the information on phenology was obtained from Waring & Townsend [44], Sterling & Parsons [45] and a local moth recording website [52].

Skews in amplification of multi-template extracts can arise due to PCR stochasticity and primer preference [50,53]. In this study, generic arthropod primers (ZBJ-ArtF1c and ZBJ-ArtR2c) amplifying a 157 bp mini-barcode fragment were used [34]. When designed and tested, feeding trials and comparisons between molecular and morphological analyses suggested that the primers did not suffer from significant amplification biases and showed that they are able to co-amplify insect DNA in mixed templates [34]. Subsequently, the primers have been used in molecular bat diet studies where they have amplified insect DNA across orders e.g. [34,36,37,54,55]. In the current study, each extract was amplified twice with a unique barcode combination and during subsequent analyses only sequences appearing in both PCRs were computationally retained. As PCR and sequencing errors arise independently, this approach was chosen in order to minimise the occurrence of artefacts in the final results. This conservative approach has the drawback that sequences originating from rare templates which might not be amplified in both PCRs due to PCR stochasticity [53,55] or templates which the primers are less likely to amplify due to primer preferences, might be discarded alongside sequences with PCR and/or sequencing errors. Therefore, many metabarcoding studies take the opposite approach when dealing with mixed templates. That is, after amplifying each extract a given number of times, all sequences originating from these amplifications are retained e.g. [56,57]. Future metabarcoding bat diet studies might benefit from a combination of these two approaches, i.e. by performing a study-dependent number (>2) of PCR replicates per extract, based on the number of replicates in which a sequence should appear, in order to be retained can be determined. This might help balance the trade-offs between detecting true biological sequences from low-templates and eliminating PCR artefacts.

Amplified Cyt b sequences were successfully obtained from 81% of extracted faecal samples. This is lower than that reported by Boston et al. [58] who successfully amplified a larger fragment size (ca. 1200) with a greater degree of success 89%. Our lower amplification success of a shorter fragment may be due to differences in extraction protocol or higher DNA degradation due to non-optimal storage conditions.

Although a correlation was apparent between the percentage frequency of prey orders in molecular and morphological analyses, some major differences in the representation of prey as revealed by these methods occurred (Table 1). For example, Isopoda were more highly represented in the molecular analysis, and arachnids and lepidopterans were relatively more represented in the morphological analysis. Both molecular and morphological methods for dietary analysis have biases [32]. Controlled feeding experiments in which bats are fed measured amounts of known prey types and their faecal droppings are analysed with both methods would be informative for better understanding these biases.

We found 99 OTUs in the bat faecal pellets of which we assigned 72 to an insect species (Table 2). Since the microscopic analysis was not able to go beyond family level, we cannot compare the molecular and morphological results at either genus or species level and without having performed controlled feeding experiments, we can only assume that the obtained number of OTUs gives an accurate representation of the insect species present in the analysed faecal pellets.

### Previous studies on the diet of M .nattereri

Previous dietary studies (See [59] for a review of studies prior to 1995; [18,60,61]) revealed that *M. nattereri* has a wide dietary niche breadth, taking prey from a diverse range of arthropod orders [59]. *M. nattereri* eats predominantly diurnal dipteran species [59,62], which presumably are gleaned from their nightly resting places [59,62]. However, these studies were based on examination of faecal pellets usually gathered from below nursery colonies between May and September [60,61,63], and none assessed the diet of *M. nattereri* during winter.

Due to the seasonal and geographic variation in the abundance of prey [60] and changes in bats’ seasonal requirements, considerable differences in diet have been observed both among study sites and at different times of the year in rhinolophid (horseshoe) bats [64,65]. In a study of the winter diet of the greater horseshoe bat (*Rhinolophus ferrumequinum*), Ransome [21] found that insects in the winter diet were broadly similar in type (but not importance) to those found in their summer diet. As during summer, there was variation in prey species taken between study sites [64]. However, prey species taken at sites (but not proportions) remained consistent across years.

### Predation of Lepidoptera

The tympanic organs of many moths appear to have evolved mainly to detect echolocating bats [66]. In free flight, *M. nattereri* emits broadband calls that pass through the frequency range where moth hearing is at its most sensitive [67]. These factors probably render *M. nattereri* poorly equipped for capturing adult tympanate moths. This is largely supported by dietary studies of *M. nattereri* during the summer where Lepidoptera make up a relatively small component (5.4% [63] and 1.2% [60]) of the diet. However, in contrast with previous studies, our findings showed that at least at this particular study site, adult Lepidoptera make a relatively high proportion (>16%) of the diet of *M. nattereri* during winter. The temperature threshold for flight in many moth species is 6-10°C [15] (though the threshold may be lower in species that fly during winter). During our study periods, ambient temperatures ranged from -13.0–18.3°C. The ability of an insect to fly occurs abruptly at a critical temperature [3]. Therefore, moths grounded by a drop in temperature to below their flight threshold may become vulnerable to gleaning bats. Furthermore, many moth species flutter their wings when warming up, and the sound of wing-fluttering is exploited by gleaning bats [68]. Together, these features may explain our finding that adult Lepidoptera make up a larger part of the diet of *M. nattereri* during winter.

Adult lepidopteran prey remains were often encountered during the morphological analysis and the molecular study identified six lepidopteran species that would have been adult when eaten during winter (Table 3)*.* These comprised two noctuid, two geometrid, one tortricid and one ypsolophid species (Table 2; 3). Both noctuid and geometrid moths are known to possess tympanic organs [42], and the geometrid winter moth *Operophtera brumata* initiates evasive flight in response to ultrasound, indicating bat avoidance behaviour [69]. However, tympanic organs have not been found in the Tortricidae [70] and there is no published work on the presence of tympanic organs in the Ypsolophidae.

Unexpectedly, we detected large quantities of Lepidopteran larvae in the winter diet of *M. nattereri*. Lepidopteran larvae have previously been recorded within the diets of bats [71-73] including *M. nattereri*[59,74]. The proportion of lepidopteran larvae recorded in the morphological analysis should be considered a conservative estimate due to a reduced likelihood of identifiable body parts of soft-bodied prey surviving passage through the bats’ digestive systems [75,76]. Many Lepidoptera overwinter in their larval stages and are active on mild winter nights [44,77], and some species are able to withstand temperatures below freezing [78-80]. Once located, caterpillars should be easily captured by a gleaning bat and provide considerable nutrition [81]. Gleaning bats may eat larvae hanging from silk threads [73] when they would be an obvious target readily detectable by echolocation [73]. It seems unlikely, though, that larvae would be hanging from threads during winter nights. We have not identified where the *M. nattereri* in our study caught the larvae identified in the diet nor the foraging strategy used to gather them. However, the larvae we encountered in the diet of *M. nattereri* do feed on mild winter nights, and they are most commonly encountered in open grassy lowland habitats [44].

Globally, bats are known to provide a range of valuable ecosystem services [82], including reducing the numbers of insect pests [83,84]. We found that Noctuidae was the most frequently encountered lepidopteran family represented mainly by the square spot rustic, *X. xanthographa,* which were identified in 80.6% of the pellets in the molecular analysis, and the large yellow underwing, *Noctua pronuba,* which were found in 63% of the pellets in the molecular analysis*. N. pronuba* larvae are often referred to as ‘cutworms’ and are considered serious agricultural and horticultural pests in Europe [85] and North America [86]. Although the total abundance of UK moths decreased by 28% between 1968 and 2007, the UK population of *N. pronuba* increased by 186% over the same period [87]. Further studies are needed to determine whether *M. nattereri* provides valuable ecosystem services by consuming *N. pronuba* and related agricultural pest species.

### The consumption of other prey species

Some arachnid species are active during winter [88,89] even at very low temperatures [90,91]. We identified 14 arachnid species in the diet of *M. nattereri*. Of these, approximately half are web-building while the others are non-web-building or hunting spiders. The species identified are found on the leaves of herbs, shrubs and trees both within the canopy and close to the ground (M. Nyffeler, personal communication). The lycosid species (*Pardosa* sp) can usually be found on the ground or on low herbs in woodlands, grassland and agricultural fields (M. Nyffeler, personal communication). Arachnids have been recorded frequently in the diet of *M. nattereri* during summer [60-62,76]. They are presumably gleaned from the ground or other surfaces, or taken directly from webs [92] where they may be obvious targets for echolocating bats. Linyphiid spiders have also been observed ‘ballooning’ in favourable weather conditions during winter [93] so the capture of some species by aerial hawking cannot be ruled out. The possibility of some arachnid species appearing in the diet as a result of secondary predation must be considered [50]. However, analysis of the contents from individual faecal samples shows that although some arachnids were found within the same sample, many were not; also morphological results confirm prey remains from different arachnid families. If secondary predation was involved, it might be expected that the prey were digested completely by the predatory spider and therefore not visible under the microscope.

Isopod fragments were recorded in both the morphological and molecular analysis. Molecular analysis identified fragments as belonging to the common striped woodlouse (*Philoscia muscorum*). Woodlouse body fragments are not commonly encountered within bat faecal remains, leading some to conclude that bats do not eat woodlice [94]. Woodlouse body parts have, however, been recorded previously in the diets of long-eared bats (*Plecotus auritus* and *Plecotus austriacus*) [37,95], *M. nattereri*[95] and the lesser horseshoe bat (*Rhinolophus hipposideros*) [22]. Woodlice are known to be active at night and remain active throughout winter [96], and they were commonly observed to be active on winter nights within our study area (P. Hope, unpublished observations).

Woodlice appeared in a higher proportion in the DNA results than in morphological findings, which may be caused by a number of factors. It is possible that faecal pellets may have been contaminated with woodlice DNA prior to collection. However, the fact that woodlouse body parts were identified in faecal pellets during the microscopic analysis, verifies that they are consumed during winter. The lower proportion of woodlouse remains identified in the microscopic analysis may be because certain body parts are discarded (e.g. the pereon) by bats due to difficulty in digestion or have low nutritional value or energy content. This is supported by the fact that we only found woodlouse leg parts, antennae and mouthparts during the microscopic analysis of the faecal pellets.

In previous studies, Diptera was the most frequently recorded order in the diet of *M. nattereri.* We identified eight species from six families and over the whole winter period, Diptera made up 11.5% (morphological) and 16.4% (molecular) of the bats’ diet. There was a tenfold decrease in Diptera between December and January-February (2%) in the morphological findings, with a less pronounced decrease in the molecular findings (18% and 13% respectively). In the particularly mild maritime climate of Cornwall, UK, Williams et al. [22] recorded Diptera as the most commonly encountered prey item over winter in the diet of the lesser horseshoe bat (*R. hipposideros*)*,* as is the case during summer [97]*.* Diptera were either less abundant during winter in our study area or *M. nattereri* may have selected alternative prey items that may be easier to capture or have greater nutritional value (e.g. Lepidoptera adults and larvae).

The majority of dipteran species identified within our study are known to be active during winter. Dung flies (*Scathophaga stercoraria*) were recorded as a dominant prey species in the winter diet of greater horseshoe bat (*R. ferrumequinum*) [21] and cluster flies (*Pollenia rudis* and *Pollenia pediculate*), which overwinter as adults, become active during spells of mild weather and are commonly found in bat roosts in the UK [98]. Also, dipteran species from the family Muscidae (of which three species were identified in our study) are active as adults during winter [99].

We identified the tachinid fly (*Ramonda spathulata*) among the prey. It is a known parasite of noctuid moth caterpillars [100]. *Ramonda spathulata* was identified molecularly in seven of the bat faecal samples and was always found in conjunction with noctuid larvae or an unidentified lepidopteran species, which suggests that it was consumed indirectly.

### Sensory ecology and its influence on foraging

The ability to detect stationary or slow moving prey on or close to surfaces using echolocation would be highly advantageous for foraging in winter when low temperatures reduce insect activity. Our finding that *M. nattereri* eats a high proportion of non-volant prey concurs with studies conducted between the months of May and September. Shiel et al. [63] concluded that 68% of the prey items eaten by *M. nattereri* were non-volant. Differences in sensory ecology among gleaning bat species contributes to niche differentiation [18]. Some gleaning bat species listen for prey-generated sound cues to detect and locate prey [101,102]. Mouse-eared bats (*Myotis myotis*) and (*Myotis blythii*) enter a ‘whispering mode’ of echolocation during the final stages of prey approach [103], while the brown long-eared bat (*Plecotus auritus*) ceases to use echolocation when attempting to capture prey in cluttered environments [61]. *M. nattereri* in contrast emits a feeding buzz [61] and uses very broadband echolocation pulses to locate immobile prey close to vegetation [104].

Some gleaning bat species appear unable to detect non-moving prey that remain on the surface of vegetation [105] while others can [106]. The detection of stationary prey amongst dense vegetation (e.g. leaf litter), however, would be more difficult due to the effects of increased acoustic clutter [107]. Some bats may be able to discern texture when using echolocation because spectral interference of echoes reflected from surfaces allows discrimination of targets [108]. It would be interesting to establish whether *M. nattereri* is able to detect sufficient textural detail with its echolocation calls to distinguish slow moving or stationary prey from background clutter. *M. nattereri* may use hairs fringing the outer margin of the uropatagium to detect prey at very close range [109], and bats of this species have been observed trawling through vegetation using their tail membrane to gather prey [61]. *Myotis nattereri* has also been recorded using quadrupedal movement on the ground to chase and capture prey [61,74]. This behaviour may also be used to locate and capture non-volant or immobile prey during winter.

## Conclusions

Until now, the winter diet of vespertilionid bats in locations with mild temperate climate has been largely overlooked. This study indicates that, at least in a location with a mild winter climate, *M. nattereri* forages throughout winter. Morphological data show that a high proportion of non-volant arthropods are taken as prey throughout the winter, while the molecular data provide a very detailed picture of the winter diet of *M. nattereri,* identifying 43 prey species from six taxonomic orders. Previous studies on the diet of *M. nattereri* in summer revealed high incidence of Diptera in the diet, and studies in a flight tent revealed that the species catches prey close to surfaces by using broadband echolocation calls. Unexpectedly, we detected large quantities of lepidopteran larvae, which must have been captured by gleaning. The high occurrence of other non-volant prey also suggests that a gleaning foraging strategy is employed during winter, which may be effective at low ambient temperatures when the abundance of flying insects may be reduced substantially.

## Materials and methods

### Study site

Our study site was in Greywell Tunnel, which is a 1,125 m-long canal tunnel on the Basingstoke Canal, Hampshire, Southern England, United Kingdom (UK) (51.266380, -0.988030 (WGS1984)). Greywell Tunnel has not been used for navigation since the 1930s when a roof fall blocked a central section [110]. Both east and west portals of the tunnel have been grilled to prevent unauthorised human access, though grilles are designed to allow bats to freely use the site. The tunnel was designated a Site of Special Scientific Interest (SSSI) in 1985 in recognition of its importance as a bat hibernation site [111].

Average, minimum and maximum daily temperature readings from December 1 to March 31 2009–10 and 2010–11 were measured at a weather station at a Royal Air Force (RAF) base (RAF Odiham) 2 km from Greywell Tunnel. Mean daily temperatures (with *SD* and range in parentheses) between December 1 and March 31 were: in 2009–10, 3.4 ±3.6°C (-8.6–16.0°C) and in 2010–11, 4.3 ±4.1°C (-13.0–18.3°C).

### Sample collection and preparation

Bat faecal samples were collected from within the western end of Greywell Tunnel during the winters of 2009–10 and 2010–11. Over the collection period, the number of visible *M. nattereri* ranged from ten (February 2011) to 63 (January 2011). A radio telemetry study conducted during 2008–09 and 2009–10 [13] showed that there were changes in the hibernating population with new individuals coming to and leaving the site over the winter. Bats are also known to roost in cavities behind the tunnel’s brick lining [25], so counts of visual bats may underestimate the total number of bats present.

Heavy-gauge white polythene sheeting (3.9 m × 4.9 m) was laid down on a clay surface close to the roof fall at approximately 130 m from the western portal. Faecal samples were collected between December-March, to minimise disturbance to hibernating bats samples were collected at the end of each month. During collection, each faecal sample was placed in a sterile 1.5 ml tube. After sample collection, the sheeting was cleared of bat faeces to avoid any mixing of faecal material between survey months. Fewer samples were collected during January and February of both study years so the samples from these months were pooled to produce a mid-winter sample size sufficiently large for comparison with December and March samples. Samples were stored at room temperature.

### DNA extraction

Fifteen droppings were selected randomly from each of the three survey periods for both study years (*n* = 90). DNA was extracted from each faecal pellet using QIAamp DNA Stool Mini Kits (Qiagen Ltd., Crawley, West Sussex, UK) following the methodology as set out by Zeale et al. [34], all extractions were conducted during a single, two week period. After DNA extraction, faecal pellet remains were placed in 2 ml tubes and frozen for later morphological analysis.

### Assigning pellets to bat species

To identify bat species to pellets, faecal extracts were amplified using forward (R3.1-F 5′- TGA AAA ACC ATC GTT GTA TTT CAA CTA CAA-3′ [112]) and reverse (MVZ04 5′-GCA GCC CCT CAG AAT GAT ATT TGT CCT C -3′ [113]) primers amplifying a 405 bp fragment of the mitochondrial DNA (mtDNA) cytochrome *b* gene. A HotStar Taq *Plus* Master Mix Kit (Qiagen Ltd., Crawley, West Sussex, UK) was used. PCR mix for each sample (20 μl) was as follows: 1 μl of extracted faecal DNA, 1 μl each of forward and reverse primers (final concentration in reaction 0.1 μM), 2 μl of loading buffer (CoraLoad), 10 μl HotStarTaq Master Mix (HotStar Taq plus DNA polymerase, PCR buffer (with 1.5 mM MgCl_2_) and 200 μM each dNTP) and 5 μl H_2_O. PCRs were carried out on a PTC-200 Thermo Cycler (MJ Research, Reno, Nevada) with the following conditions: 95°C for 5 minutes followed by 34 cycles of 94°C for 30 seconds, 50°C for 30 seconds, 72°C for 1 minute, followed by a final extension at 72°C for 10 minutes. Six blank negative controls were included in the PCR. PCR products were visualised on a 1.5% agarose gel stained with Web Green DNA stain (Web Scientific). Sanger sequencing of PCR products was performed in one direction (using the primer MVZ04) and carried out by a commercial laboratory (LGC Genomics, Germany).

Sequences were compared with reference sequences deposited in GenBank using the Basic Local Alignment Tool (BLAST) to obtain ‘closest match’ identifications. Matches were classed as positively identified to bat species if maximum identity was at ≥98% and query coverage ≥70%.

### Morphological analysis

Morphological analysis of faecal material was conducted using traditional microscopic methods [32,63,76]. Faecal material was re-suspended in a 90 mm diameter Petri dish; a 2x2 mm piece of graph paper was fixed to the bottom of the dish to aid judgement of fragment size. One drop of glycerol, then one drop of alcohol (70%) were added to help spread prey fragments evenly over the Petri dish. Fragments were examined using a binocular microscope (MX3, 20x and 40x magnifications, Brunel, Chippenham, Wiltshire, UK). All fragments considered suitable for identification were mounted on a microscope slide in glycerinated gelatine under an 18x18 mm cover slip.

Arthropod fragments were compared with examples in published keys or identification guides [76,114] and attempts were made to identify fragments to insect order, or where possible family. The relative importance of different prey orders in the diet was quantified in two ways. First, we calculated the percentage occurrence (%O) of prey orders, which is the number of pellets that an order was found in / total number of pellets in samples x 100 [76]. Second, we calculated the percentage frequency (%F), which is defined as the number of occurrences of an order divided by total occurrences of all orders x 100 [76]. Whereas %O produces values that exceed 100% when all taxa are combined (as pellets can contain more than one taxon), %F gives a proportional representation of the relative importance of prey taxa rounded to 100%.

### Molecular analysis

In order to identify insect-prey in the bat droppings, amplifications of ca. 157 bp prey-insect-COI fragments were performed on the faecal extracts following a modification of the methods described in Bohmann et al. [36]. The assay described here was customised for Illumina MiSeq sequencing as opposed to GS FLX sequencing used by Bohmann et al. [36]. Specifically, insect-generic COI mini-barcode primers (ZBJ-ArtF1c and ZBJ-ArtR2c [34] were 5′ nucleotide barcoded [115], yielding a set of 20 forward and 20 reverse primers, all varying by the 8 bp of the 5′ barcode (Additional file 1), producing a final amplicon size of 227 bp (157 bp insert, 70 bp primers). Subsequently, all PCRs performed on the DNA extracts were performed using different combinations of the 5′ barcoded forward and reverse primers, ensuring that all resulting positive amplicons from each PCR reaction were uniquely labelled. To aid downstream bioinformatics processing of the data, and in particular to enable discrimination of PCR and/or sequencing artefacts from true biological sequences, each extract was subjected to PCR twice, in each case incorporating a unique barcode combination to each reaction. PCR reactions were performed in 25 μl reactions using the Amplitaq Gold enzyme system (Roche, Basel, Switzerland). For every eight reactions, a PCR blank was included as negative control. Furthermore, extraction blanks were amplified. Each 25 ul reaction contained: 1 μl DNA, 1x PCR Gold buffer, 2.5 mM MgCl_2_ solution, 0.2 mM dNTPs, 0.2 μl AmpliTaq Gold, and 0.4 μM of each primer. Amplifications were performed on a 2720 Thermal Cycler (Applied Biosystems) with the following conditions: 95°C for 5 minutes, then 40 cycles of 95°C for 15 seconds, 52°C for 30 seconds and 72°C for 30 seconds, followed by a final extension at 72°C for 7 minutes. 5 μl of each of the PCR products were transferred to a tube under a flow hood before being visualised with GelRed Nucleic Acid Stain (Biotium) on 2% agarose gels against a 50 bp ladder. Negative controls were negative and were not included in further analyses.

All PCR products were pooled into a single pool before library building, at approximately equimolar ratios (as determined by gel band strength). Specifically, bands were visually discriminated into three categories: bright, semi-bright and faint, from which PCR products were added to the pool with 1 μl, 5 μl and 15 μl, respectively. The pools of PCR products were purified using the MinElute PCR Purification Kit (Qiagen) following the MinElute Handbook 03/2008 MinElute PCR Purification Kit Protocol (using a microcentrifuge) with the following modifications: in step 4, the sample was centrifuged at 6000 g. In step 6, 700 μl Buffer PE was added, and the column was centrifuged at 10,000 g. In step 7, the empty column was centrifuged at 13,000 g for 2 minutes. In step 9, after Buffer EB was added to the centre of the membrane, the column was incubated for 5 minutes at 37°C. The column was centrifuged for 1 minute at 13,000 g, and the tube was turned and the spin repeated before DNA was collected.

The purified PCR pool was size-selected (227 bp +/-10%) on a LabChip XT (Caliper), before being purified using MinElute columns following the above-mentioned protocol and eluted in 42.5 μl Qiagen Buffer EB. The concentration of the purified, size-selected PCR pool was measured using a Qubit Flourometer (Invitrogen). The pool was subsequently converted into an Illumina sequencing library, using the NEBNext DNA Library Prep Master Mix Set for 454 (#E6070L) although using blunt end Illumina adapters [116] in place of Roche/454 FLX adaptors. The NEBNext End Repair Module Protocol was followed with the following modifications: end repair was performed in a 50 ul reaction using 42.5 μl size-selected, purified PCR pool. A control library blank was constructed in which 42.5 μl H_2_O was added in place of DNA. They were incubated in a 2720 Thermal Cycler (Applied Biosystems) for 20 minutes at 12°C and 15 minutes at 37°C, before being purified on MinElute columns following the above-mentioned protocol. In the Quick Ligation Module procedure, 0.5 μM Illumina sequencing adaptors [116] were included in the 50 μl reaction, and the mix was incubated on a 2720 Thermal Cycler (Applied Biosystems) for 20 minutes at 20°C before being purified on MinElute column following the above-mentioned protocol and eluted in 42 μl Buffer EB. In the fill-in reaction procedure, only step 8 was performed in which 5 μl Adapter Fill-in Reaction Buffer and 3 μl Bst DNA polymerase was added to the 42 μl DNA eluted above. This was incubated in a 2720 Thermal Cycler (Applied Biosystems) for 20 minutes at 65°C, followed by 20 minutes at 80°C to inactivate the enzyme.

The library was subsequently subjected to index PCR in 50 μl reactions using the Amplitaq Gold enzyme system (Roche, Basel, Switzerland): the library was amplified in five separate reactions each containing 5 μl library, alongside a reaction containing 5 μl of the library blank and one PCR blank in which 5 μl H_2_O was added. Furthermore, each reaction contained 1x PCR Gold Buffer, 2.5 mM MgCl_2_ solution, 0.25 mM dNTP’s, 5x purified BSA (BioLabs), 0.4 μl AmpliTaq Gold, 29.6 H2O, 0.2 μM forward index primer paired end (InPe 1.0) and 0.2 μM reverse index primer paired end. The five reactions containing the library were amplified with the same reverse index, while the library blank and the PCR blank were amplified with different reverse index primers. The index PCR was run on a 2720 Thermal Cycler (Applied Biosystems) at the following conditions: 95°C for 5 minutes, then 15 cycles of 95°C for 30 seconds, 60°C for 30 seconds and 72°C for 30 seconds, followed by a final extension at 72°C for 7 minutes. The five 50 μl indexed libraries were combined to a total of 250 μl before the library, library blank and PCR blank were purified on Qiagen QIAquick columns following the above-mentioned MinElute purification protocol and eluted in 30 μl Buffer EB. The library, the library blank and the PCR blank were visualised on a 2100 Bioanalyzer (Agilent Technologies). As no target fragments were detectable in either the library blank or the index PCR blank, only the library was sequenced. Sequencing was performed on the Illumina MiSeq platform where tagged amplicons were sequenced 150 bp paired-end, taking up ca. 15% of a run.

Initial bioinformatics analysis was conducted using the Quantitative Insights Into Microbial Ecology software package (QIIME) [117]. First, the quality of the paired-end reads was assessed with FastQC v 0.10.0 [118] and then low quality bases were trimmed at the 3′ end (q<20) with PRINSEQ v0.15 [119]. Although q<30 is commonly used in studies with modern DNA (good quality material), the DNA in our study showed more damage so we used q<20 which is normally used for ancient DNA which is more degraded. Afterwards, using customized Perl scripts (program available upon request) the corresponding paired reads (363,844) were merged if the overlap was 100% identical producing 140,009 sequences. In order to discriminate PCR and sequencing artefacts from true biological variation, only sequences present with 100% identity in both PCRs from the same extract were kept; this step yielded 114,345 sequences.

Also, the sequences were collapsed so that each sequence was only present once and reads shorter than 157 bp were filtered out. This gave a final filtered total of 1130 unique sequences that were used for the rest of the pipeline.

COI barcode sequences from different taxonomic levels (Arachnida, Chilopoda, Insecta, and Isopoda) were downloaded from the BOLD v3 [120] Public Data Portal to create a database and its corresponding taxonomy map to be used for QIIME v1.6.0 [117].

Species identification was made with QIIME with the uclust_ref OTU (Operational Taxonomic Unit) picking method (uclust v1.2.22q), using 98.5% identity (two mutations), with max_accepts and max_rejects set to 0 to force a search of the entire database with every query, thus guaranteeing that the best hit will be found if one exists. The biom table obtained from the OTU picking was summarised through plots with QIIME. 267 sequences failed to be assigned to a cluster (11%), and 2162 (89%) were assigned an OTU.

After the sequence data were obtained, we made the taxonomic assignments. OTUs were matched to most likely arthropod species using the identification tool within the BOLD database, using four confidence levels based on sequence similarity largely following Razgour et al. [37], whereby confidence level 1 = solid match to one species or genus (>98.5% sequence similarity); level = 2 match to more than one species (98.5%), only one of which was a UK species; level 3 = matched to two UK species of the same family (>98.5%) and level 4 = match to several species of different genera, or to reference sequences only identified to family (>98%). See Zeale et al. [34] for justification of the use of these levels of sequence similarity to taxonomic levels. As *Noctua pronuba* and *Noctua janthe* only differ by one base pair yielding a 99.4% similarity, these were clustered together at level 3. Since *Noctua pronuba* is of special interest due to its status as a serious agricultural and horticultural pest [85,86], these sequences were separated at 100% similarity to identify the extent to which *M. nattereri* had preyed on *Noctua pronuba* at this site.

Field guides [44,45,77] and information from local records [52] were used to ascertain the phenology and life cycle stage of lepidopteran species identified genetically within the diet (Table 3).

## Competing interests

The authors declare that they have no competing interests.

## Authors’ contributions

PRH conceived the study, conducted the morphological analysis, DNA extraction, amplification of bat DNA and drafted the manuscript. KB conducted the amplification and second generation sequencing of arthropod DNA and drafted part of the manuscript. MTPG supervised the amplification and second generation sequencing of arthropod DNA and commented on the manuscript. MLZM conducted the initial bioinformatics analysis of the second generation sequencing results and drafted part of the manuscript. OR assisted with extraction and PCR of bat DNA and commented on the manuscript. GJ conceived and supervised the study and assisted in drafting the manuscript. All authors read and approved the final manuscript.

## Supplementary Material

Additional file 1 **Zbjart_short primers. **xls. Primers. 20 forward and 20 reverse primers, Illumina sequencing. Click here for file
